# Reduced uptake of the proliferation-seeking radiotracer technetium-99m-labelled pentavalent dimercaptosuccinic acid in a 47-year-old woman with severe breast epithelial hyperplasia taking ibuprofen: a case report

**DOI:** 10.1186/1752-1947-4-89

**Published:** 2010-03-17

**Authors:** Vassilios J Papantoniou, Evangelia K Sotiropoulou, Pipitsa N Valsamaki, Angeliki G Tsaroucha, Maria G Sotiropoulou, Nikolaos D Ptohis, Aikaterini J Stipsanelli, Konstantinos E Dimitrakakis, Spyridon G Marinopoulos, Spyridon T Tsiouris, Aris J Antsaklis

**Affiliations:** 1 Department of Nuclear Medicine, Alexandra University Hospital, Vasilissis Sofias Avenue, Athens, 11528, Greece; 2 Department of Radiology, Sotiria General Hospital, Mesogeion Avenue, Athens, 11527, Greece; 3 Department of Pathology, Alexandra University Hospital, Vasilissis Sofias Avenue, Athens, 11528, Greece; 4 Department of Radiology, Alexandra University Hospital, Vasilissis Sofias Avenue, Athens, 11528, Greece; 5 Department of Gynaecology and Obstetrics, Alexandra University Hospital, Vasilissis Sofias Avenue, Athens, 11528, Greece

## Abstract

**Introduction:**

Recent studies have reported a risk reduction in the progression of benign breast disease to breast carcinoma through COX-2 pathways.

**Case presentation:**

We present a case of severe epithelial hyperplasia in a 47-year-old woman with increased breast density submitted to scintimammography by the proliferation-imaging tracer Technetium-99m-labelled pentavalent dimercaptosuccinic acid, before and after an oral ibuprofen treatment for 4 weeks. The radiotracer uptake after ibuprofen intake was significantly reduced, both visually and by semi-quantitative analysis, based on a calculation of lesion-to-background ratios.

**Conclusion:**

In proliferating breast lesions, scintigraphically displayed reduction in Technetium-99m-labelled pentavalent dimercaptosuccinic acid uptake may indicate inhibition by ibuprofen in the pathway of malignant epithelial cell transformation.

## Introduction

Several epidemiological and laboratory studies suggest that non-steroidal anti-inflammatory drugs (NSAIDs) may have chemo-preventive effects in breast cancer, owing to their activity against cyclo-oxygenase-2 (COX-2), the rate-limiting enzyme in the prostaglandin cascade [1]. Recent studies have suggested that inflammation through COX-2 pathways may play a role in the progression of benign breast disease to breast carcinoma, and that aspirin may reduce this risk in women with similar lesions [2]. Significant reductions in the risk of malignant transformation have been reported with selective COX-2 inhibitors, as well as with over-the-counter non-steroidal anti-inflammatory drugs, including ibuprofen and naproxen [1].

Another important related consideration is the postulated association between benign proliferating breast disease, mammographic density, and subsequent malignant transformation [2-4]. Technetium-^99m^-labelled pentavalent dimercaptosuccinic acid (^99m^Tc-(V)DMSA) is a tumor-seeking radiotracer. Its relationship to focal adhesion kinase (FAK) activation and cellular proliferating activity has been described in previous reports not only for invasive but also for pre-invasive and benign proliferating breast lesions [5-8]. This case report was undertaken to investigate whether a reduced rate of cellular proliferation, mediated by ibuprofen as described in previous retrospective studies, could be visualized by alterations in the patient's ^99m^Tc-(V)DMSA uptake ratio.

## Case presentation

**Figure 1:**
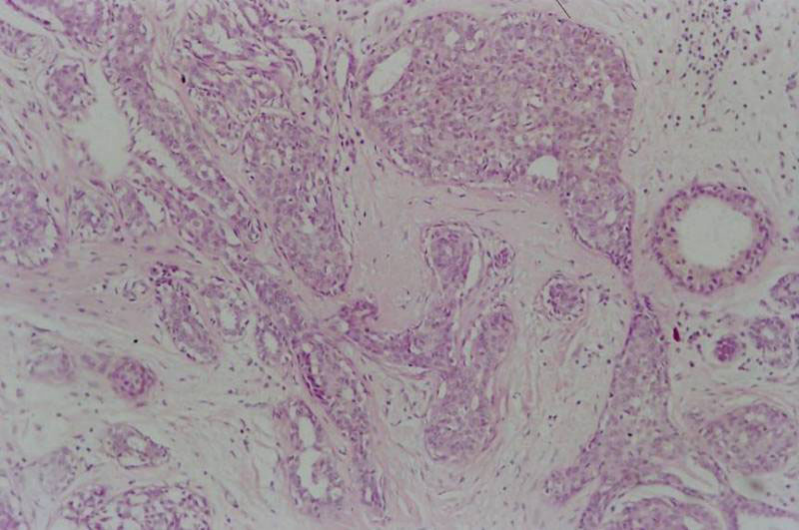
**Extensive severe ductal epithelial hyperplasia of usual type and apocrine metaplasia (Hematoxylin and Eosin staining, ×100)**.

A 47-year-old Caucasian woman of Greek national origin was referred to our department with a mammogram showing increased breast density with multiple dispersed nodular opacities, linear opacities, periareolar fibrosis, and microcalcifications in the lower outer quadrant of her right breast. Histology of an open biopsy specimen (Figure 1) showed foci of severe epithelial hyperplasia, areas of calcification, and apocrine metaplasia. Scintimammography with ^99m^Tc-(V)DMSA was performed 2 days before the scheduled biopsy and then 7 months later. Within 4 weeks before the follow-up study, our patient took oral ibuprofen (400 mg daily) for persistent musculoskeletal back pain.

After intravenous administration of 925MBq of the tracer, early and late planar (lateral prone and anterior supine) images were acquired at 10 minute and 60 minute after injection. Breast ^99m^Tc-(V)DMSA uptake in the early and late images was evaluated visually. Quantitative comparisons between the 10 minute and 60 minute scans and between the baseline study before biopsy and after the course of ibuprofen were performed by drawing regions of interest (ROIs) over the breast sites of greatest tracer uptake and over the normal breast parenchyma. The lesion-to-background (L/B) ratios were then calculated and compared between the same corresponding breast areas in the two scintigraphic studies.

**Figure 2:**
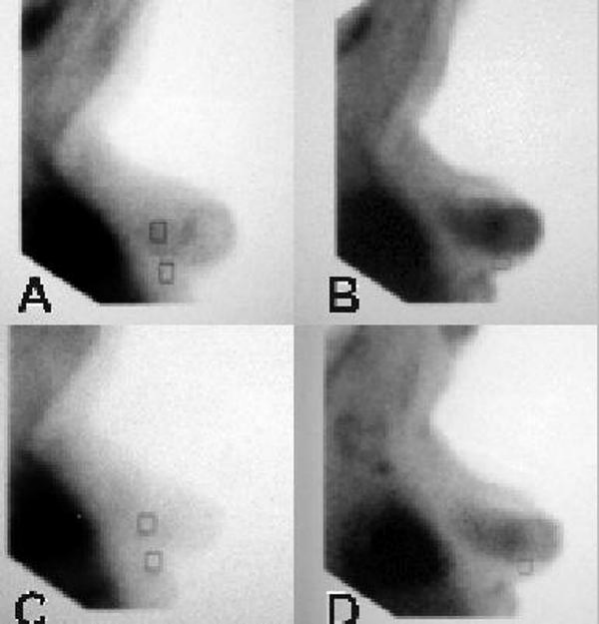
**Images of (A) early pre-ibuprofen, (B) late pre-ibuprofen (C) early post-ibuprofen, and (D) late post-ibuprofen treatment of ^99 m^Tc-(V)DMSA distribution**. After ibuprofen treatment, the diffuse tracer uptake is clearly diminished in both early (C) and late imaging (D).

A pattern of diffuse widespread tracer uptake corresponding to pre-invasive breast pathology (epithelial hyperplasia and *in situ *carcinoma, according to our previous reports [5,8]), was also observed in this case (Figure 2). This diffuse ^99m^Tc-(V)DMSA distribution almost entirely occupied our patient's right breast parenchyma and was evident in the images both before (Figures 2A and 2B) and after her ibuprofen treatment (Figures 2C and 2D). There was a gradual increase in the relative uptake of the tracer on the delayed images, compared with the early ones. However, after her ibuprofen treatment, diffuse tracer uptake was clearly diminished in both the early (Figure 2C) and late (Figure 2D) images. The L/B ratios in the 10 minute and 60 minute images were 1.562 and 2.719 (Figure 2A and 2B, respectively) in the baseline study versus 1.229 and 1.993 (Figure 2C and 2D, respectively) at follow-up examination. Based on our recent study, women without epithelial hyperplasia or with usual ductal breast hyperplasia without increased cellular proliferation rate (Ki-67 ≤ 3%) show ^99m^Tc-(V)DMSA L/B_60 min _ratios in the range of 1.07 to 1.31 (mean = 1.15) and 0.77 to 1.62 (mean = 1.2), respectively (5).

## Discussion

Our key finding is that a short period of ibuprofen treatment resulted in a 27% reduction in the uptake of ^99m^Tc-(V)DMSA in a case of proliferative benign epithelial breast hyperplasia. Other recent studies have shown that COX-2 inhibitors may reduce the risk of breast cancer [1]. Specifically, a retrospective study of almost 1000 women showed that a selective COX-2 inhibitor, celecoxib 200 mg/day for at least two years, reduced the risk of breast cancer by 83%, while rofecoxib 25 mg/day reduced the risk by 64% [1]. The non-selective COX inhibitors aspirin and ibuprofen and/or naproxen gave a reduced odds ratio (0.49 and 0.37, respectively, with 95% confidence intervals) for the incidence of breast cancer compared with non-use. Moreover, the odds ratio for breast cancer by dose and frequency was 0.28 for ibuprofen 200 mg more than 3 times weekly. In this context, other investigators have suggested that inflammation mediated through COX-2 pathways may play a role in the progression of benign breast disease to carcinoma, and that aspirin may reduce such risk in women with benign breast disease [2].

^99m^Tc-(V)DMSA is a tumor-seeking tracer whose cellular uptake is linked to FAK activation and cell proliferation, which is a precocious stage of malignant transformation [6,7,9]. Compared with invasive lesions, the exact mechanism of ^99m^Tc-(V)DMSA accumulation in benign proliferating diseases and in some non-proliferating diseases with higher L/B ratios is not yet clear [5,7]. Given that benign proliferating diseases generally have lower proliferation rate than invasive cancers, this raises the suspicion that ^99m^Tc-(V)DMSA reflects an earlier cell activation status of phosphorylated FAK in the process of increasing the rate of cell proliferation [5,6,8]. Hence, in our case, the reduction in diffuse ^99m^Tc-(V)DMSA uptake after a relatively short period (4 weeks) of ibuprofen treatment may indicate a "switch off" mechanism on activated FAK, rather than a slowing down of the proliferation rate. Although we have provided no biopsy confirmation after treatment, ibuprofen was the only treatment that our patient took between her scintimammographic studies. The biokinetic characteristics of ^99m^Tc-(V)DMSA support our suggestion that the observed reduction in its uptake was attributable to ibuprofen-induced cyclo-oxygenase COX inhibition.

## Conclusion

Our research so far has shown that diffuse tracer uptake during ^99m^Tc-(V)DMSA scintimammography can be considered indicative of an underlying proliferative hyperplastic or *in situ *pathology. This report focuses on a patient with severe breast epithelial hyperplasia enrolled in a current prospective study. In another recent report on hyperplastic lesions [10], we studied the imaging properties and biokinetic characteristics of ^99m^Tc-(V)DMSA in relation to mammographic density. As long as these lesions can be visualized, it would be of great clinical interest if we could estimate the effectiveness of various chemopreventive agents by quantifying their effect on ^99m^Tc-(V)DMSA uptake.

## Consent

Written informed consent was obtained from the patient for publication of this case report and any accompanying images. A copy of the written consent is available for review by the Editor-in-Chief of this journal.

## Competing interests

The authors declare that they have no competing interests.

## Authors' contributions

VP conceptualized the case report, contributed substantially to the organization of the performance of the relevant scintiscans described in this report, and wrote parts of the manuscript. All of the authors cooperated in the patient's care and participated actively in writing the manuscript. ES and NP performed and analyzed the patient's mammographic examination. PV, AT, AS and ST conducted and evaluated the scintimammographic studies. MS provided the histologic evidence. KD, SM and AA were the clinicians who followed-up the patient and referred her for further investigation. All authors read and approved the final manuscript.
